# Influence of Linseed Oil Varnish Admixture on Glauconite Clay Mortar Properties

**DOI:** 10.3390/ma13235487

**Published:** 2020-12-02

**Authors:** Przemysław Brzyski, Magdalena Grudzińska

**Affiliations:** Faculty of Civil Engineering and Architecture, Lublin University of Technology, 40 Nadbystrzycka Str., 20-618 Lublin, Poland; m.grudzinska@pollub.pl

**Keywords:** clay mortar, glauconite, linseed oil varnish, mechanical properties, water resistance

## Abstract

Raw clay is used nowadays in construction as a component of mortars and plasters and as a binder in composites based on straw or shives. It is a material with good sorption properties and vapor permeability, but it is susceptible to shrinkage, is not resistant to water, and also is characterized by low mechanical strength, which makes it impossible to be used, for example, in external plasters. Various additives and admixtures are used to improve selected properties of clay mortars. The article presents the research results and assessment of the effect of glauconite clay mortar modification with an admixture of linseed oil varnish on selected properties. Admixtures in the amounts of 1%, 2%, and 3% in relation to clay weight were used. Flexural and compressive strength, water resistance, shrinkage, drying capacity, density, and porosity of mortar, were tested. The admixture of linseed oil varnish in the amounts used in the investigation had a positive effect on some of the tested properties; regardless of the quantity of the admixture, the modified mortars had better parameters concerning flexural strength, shrinkage reduction, and water resistance than the reference mortar, without admixture.

## 1. Introduction

Clay is one of the oldest building materials, commonly used in hot and temperate climate regions. It was applied widely as mortar and plaster binder or as a component of bricks and earth walls from 7000 B.C. until the present [1]. Clay composites have many advantages, such as a good accumulation of heat (helping to reduce overheating in hot regions and lower the energy use for heating in colder regions) [1], the capability of regulating moisture content in rooms [2,3,4], the possibility of recycling, and low extraction and transportation costs [1,5]. It can be used for protection purposes in timber constructions, increasing the resistance to biodegradation of the latter [6].

The growing popularity of sustainable, ecological buildings has increased the interest in earth mortars based on natural, raw materials with low carbon footprints, such as clay and lime [7,8]. However, the main problems limiting the wide adoption of these products in building practices are:

1. Low mechanical strength [5,9,10,11];
2. Shrinkage during the drying up process [5,12,13];
3. Poor resistance to water penetration [14,15,16];
4. Low durability connected with poor frost resistance [9,16,17].

In order to enhance the properties of mortars, many studies considered the influence of various additives and admixtures on the characteristics of fresh mixtures or hardened materials. In historic, vernacular architecture, for example, there are references to the use of organic components improving the mechanical properties, workability, and waterproofing of natural mortars [6,12,15]. Some of them have been recently rediscovered and investigated [18], including, among others, sheep wool [9,19], animal glue [20], casein [20,21,22], eggs [21], olive oil [20], linseed oil [22,23,24,25], sunflower oil [26,27], straw [14,28,29,30], and hemp [31,32]. 

Inorganic substances, such as metakaolin [33,34,35,36], volcanic ash [37,38] and brick dust [35], because of their pozzolanic activity, can be, as well, used to improve the mechanical strength of cement-based and natural mortars. They can also diminish the shrinkage of the paste and mortar [33,35] and improve durability by reducing the liquid water transport [39]. 

Also man-made, processed supplements are examined as modifiers, affecting the properties of traditional building elements. The exemplary products mentioned in the literature are fly ashes [40], silica and alumina nanoparticles (stabilizing the material by reducing the shrinkage and increasing its early strength) [5], and aerogels (intended to increase the thermal resistance of the plaster) [41].

As the additives used in the presented research are meant to improve the resistance to water of the mortar, the description of the water-repellents will be extended. Among many substances, fatty acids are commonly used for this purpose. Linseed oil positively affects the impermeability and enhances the freezing and thawing resistance, thanks to the reduction in the capillary water absorption [17,23,42]. It also prevents crack development during curing [17] and reduces shrinkage as an effect of fostering the pozzolanic reaction [24]. The application of olive oil as an additive described in [20] reduced the pore size and lowered the water absorption of the mortar. Similar effects were obtained using oils from sunflower, peanuts, and rapeseeds [27]. The addition of spent cooking oils in the lime mortars produced a significant hydrophobic effect, diminishing capillary water transport [26]. 

The adverse consequences of such modification may be slower development and lower value of mechanical strength, as described in [23,25]. In some cases, however, the addition of fatty acids to the lime mortars significantly increased their hydrophobicity without worsening mechanical strength [20,26].

Some water-repellent admixtures (produced by the chemical industry and typically used in cement-based mortars), namely sodium oleate, calcium stearate, or zinc stearate, are also investigated in lime-based mortars [43,44,45]. These substances decreased the water absorption level of analyzed material, without being highly detrimental to mechanical strength. 

The hygrothermal performance of natural plasters can be also improved thanks to the application of finishing layers based on cellulose, casein, or lime [46]. However, as clay plasters are often used as the finishing of the stud walls insulated with hemp–lime composite [47,48], it is important not to limit excessively their vapor permeability.

As presented above, there are many publications concerning the additives and admixtures that improve the durability and impermeability of mortars. However, no similar research on clay mortars can be found and this paper intends to complement this research area. It presents the results of studies on the application of local clay as a mortar binder, modified by the linseed oil varnish admixture. The examined clay has a high glauconite content, giving it a greenish tinge, and the use of this specific type of binder in building mortars has not been investigated before. One of the leading applications of glauconite is as a filter component for heavy metals’ removal. As glauconite is a clay mineral and the clay mixture used was rich in loam fraction and showed a high binding capacity, the resulting admixture may have potential use as a building material. Selected properties of the obtained material, i.e., flexural and compressive strength, water resistance, shrinkage, drying capacity, density, and porosity, were tested.

## 2. Materials and Methods 

### 2.1. Mortar Recipes

Four clay mortar recipes with a variable amount of linseed oil varnish admixture were examined in the work. They were marked as CM-0, CM-1, CM-2, CM-3, where CM means “clay mortar” and the number stands for the percentage of linseed oil varnish in relation to the clay weight. All the mortars had a clay to sand weight ratio of 1:4, while the water to clay weight ratio was 0.8 (lower values of the water to binder ratio did not ensure proper workability of the mortar). The recipes used in the investigation are shown in Table 1.

**Figure 1:**
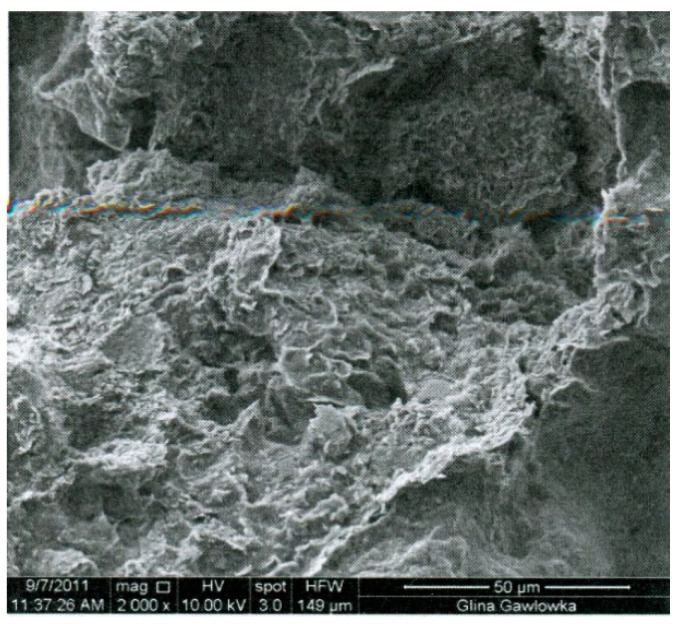
The microstructure of the examined clay [49].

**Figure 2:**
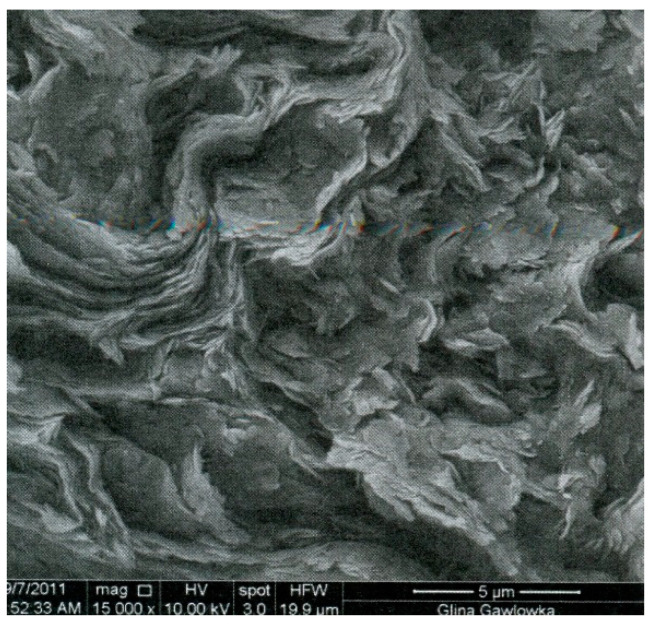
Clay minerals’ habit in the tested clay [49].

As a binder, the clay containing glauconite was applied. The raw clay material came from the sand mine in Gawłówka, Poland. The clay extracted from the mine included the sand fraction (0.05–2 mm) in the amount of 60%, the silt fraction (0.002–0.05 mm) in the amount of 3%, and the loam fraction (<0.002 mm) in the amount of 31% [49]. The sand fraction was separated, leaving a mixture of dusty and clay fractions. Glauconite is a soft mineral and its hardness is similar to that of mica minerals or slightly lower, usually around 2 on the Mohs scale. The specific density of clay was 2.68 g/cm^3^ and the bulk density of the soil skeleton was 1.98 g/cm^3^. The chemical composition of the glauconite clay used is shown in Table 2, the microstructure image is shown in Figure 1 and the clay minerals habit is shown in Figure 2. Table 2 and Figure 1 and Figure 2 come from the publication [49] and relate to the characteristics of the glauconite clay used in this study.

Quartz sand with a fraction of 0–2 mm acted as a mortar filler. Linseed oil varnish in the form of a viscous liquid with a density of 900‒940 kg/m^3^ was used as an admixture, intended to improve the aforementioned properties of the examined material. Its viscosity, measured with a Ford cup 4 mm at 20 °C, was 20–26 s.

### 2.2. Preparation of Mixture and Specimen

Because of the different working properties of fresh clay mortar compared to lime or cement mortar, the procedure of mixture preparation was not based on existing standards. Before preparing the mortars, the hardened clay was dried, crushed into smaller pieces with a hammer, and then placed in pots with water. Part of the water (by mass ratio water:clay = 0.5) was added to the weighed amount of clay according to the recipe, and then the mixture was weighed and soaked to allow the clay to soften for about 12 h. After this time, the vessels with clay and water were reweighed. The remaining water was added, supplemented by the heated linseed oil varnish admixture. Heating of the varnish was conducted in order to liquefy it so that the substance could distribute more homogeneously in the mixture. Finally, the sand was added and the composite was mixed for 3 min with a mechanical agitator until a homogeneous material was obtained. The ready mortar was formed into moulds of 40 mm × 40 mm × 160 mm and stored in dry air conditions (temperature around 20 °C, relative humidity around 50%). After 5 days of ripening, the samples were demoulded and left under the same conditions until air-dried.

### 2.3. Research Methods

#### 2.3.1. Apparent Density, Specific Density, and Total Porosity

The apparent density test was carried out according to standard PN-EN 1015-6 [50] on three cubic specimens from each recipe. The specimens of known volume were dried and then weighed on a laboratory scale to calculate the apparent density. The specific density of mortars (the ratio of sample mass to volume excluding pores) was determined by the pycnometric method based on the PN-EN 1936: 2010 standard [51]. A sample of the composite to be tested was pulverized to the fraction below 0.063 mm by milling in a ball mill (Fritsch, Idar-Oberstein, Germany). Based on the specific and apparent density results, the total porosity was calculated as the percentage ratio of the open and closed pore volume to the total sample volume. 

#### 2.3.2. Resistance to Water

To determine the destructive effect of water on the material, the resistance to water test was carried out on samples measuring 80 mm × 40 mm × 40 mm. Before the test, the samples were dried to constant weight in a laboratory drier at 80 °C. Twelve samples from each recipe (48 in total) were used for the experiment. For each time step, three samples from each recipe were tested. The course of the study was as follows:

1. Three samples for each recipe (formerly dried to constant weight) were placed in a bath of water, so that they were completely submerged, for 1 min, then taken out and dried again to constant weight;
2. After this first immersion stage, significant losses were noticed in the CM-0 samples, so it was decided to reduce the duration of the following immersion periods for the samples without varnish; thus, in the next three stages they were dipped for the periods of 5 s, 15 s, and 30 s, according to the above procedure;
3. The duration of the immersion time for the samples with varnish instead, was maintained at 3, 5, and 7 min, since no extreme reduction was noticed in these samples;
4. The same procedure explained at point 1 was repeated for the duration mentioned at point 2 (for CM-0 samples) and point 3 (for CM-1, CM-2, and CM-3 samples).

The experiment made it possible to calculate the percentage weight loss of the samples, as the relative difference between the weight of the dried sample before and after the water immersion.

#### 2.3.3. Shrinkage and Weight Loss on Drying

The susceptibility of mortars to shrinkage was determined by measuring the length of the samples immediately after forming and after complete drying. As before, three samples from each recipe were tested. The length of the samples was measured before moulding (mould length), and after 3, 6, 8, 10, 12, 15, and 19 days. During the length measurements, the samples were weighted to check their drying capacity and specify the weight loss on drying.

#### 2.3.4. Flexural Strength

The flexural strength of the mortar samples (stored for 28 days in air-dry conditions) was determined following PN-EN 1015-11 standard [52] using the MTS 809 press (MTS System Corporation, Eden Prairie, MN, USA) on 40 mm × 40 mm × 160 mm trabeculars, under the movement of the press head of 0.2 mm/min. Four samples from each of the recipes were tested, but in the case of CM-0 and CM-2, single samples were incorrectly destroyed, and the results were rejected.

#### 2.3.5. Compressive Strength

The compressive strength of the of the mortar samples (stored for 28 days in air-dry conditions) was determined using the MTS 809 press on the broken half samples from flexural strength test following the PN-EN 1015-11 standard [52]. The displacement velocity of the press head was set at 8 mm/min. The test was conducted on six samples of each recipe.

## 3. Results

### 3.1. Apparent Density, Specific Density, and Total Porosity

Apparent density, specific density, and total porosity values are shown in Table 3. 

### 3.2. Resistance to Water

**Figure 3:**
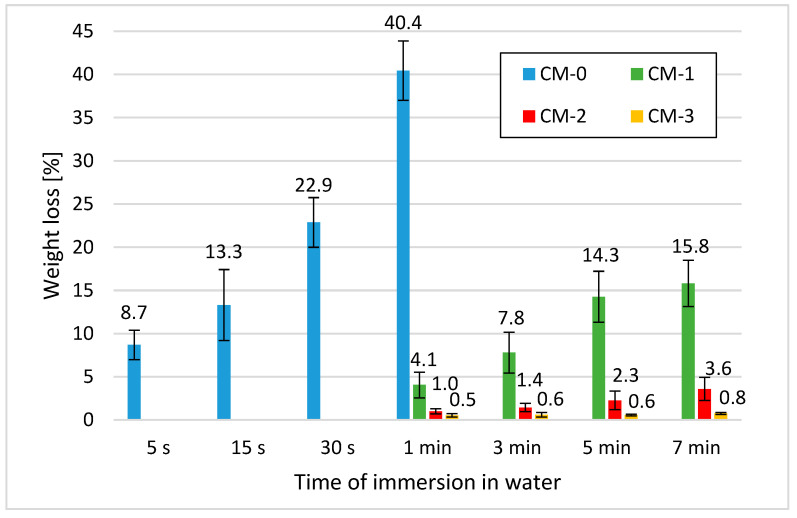
Weight loss of mortar samples after water immersion for a specified time.

**Figure 4:**
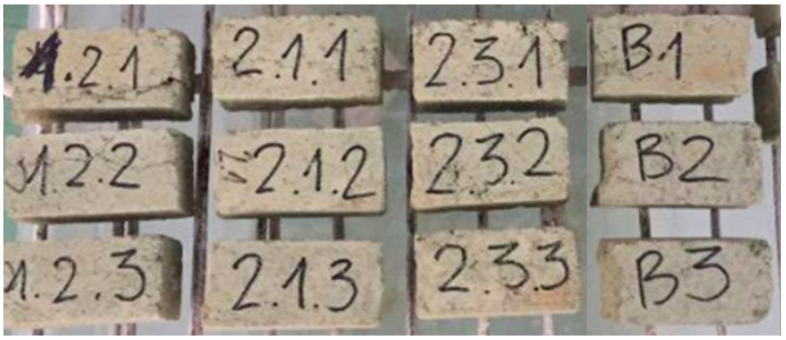
Glauconite clay mortar samples before the water immersion. From the left: CM-1, CM-2, CM-3, CM-0.

**Figure 5:**
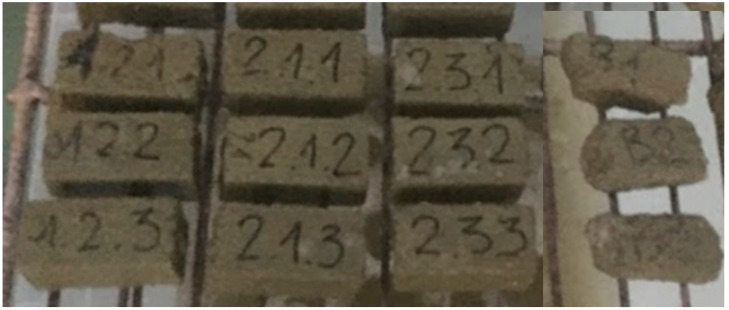
Glauconite clay mortar samples after 1 min of the water immersion. From the left: CM-1, CM-2, CM-3, CM-0.

The results of the resistance to water test are shown in Figure 3. Error bars show standard deviation. Figure 4 shows the samples before they were placed in water, while Figure 5 shows the samples soaked in water for 1 min.

### 3.3. Shrinkage and the Weight Loss on Drying

**Figure 6:**
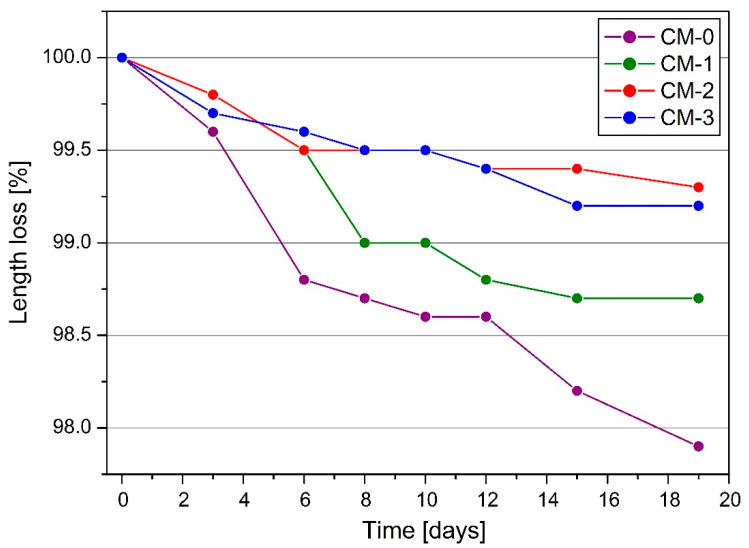
The average decrease in sample length on individual days.

The average values of the length loss of the samples as a result of shrinkage and the standard deviation are shown in Table 4, and for better visualization of the results, the trends of the length loss over time are shown in Figure 6.

**Figure 7:**
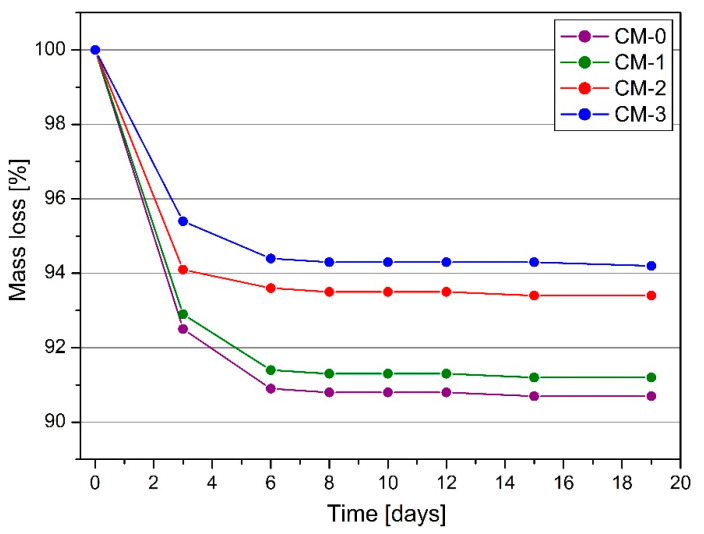
The average decrease in sample mass on individual days.

The average values of the mass loss of the samples over time and the standard deviation are shown in Table 5, while for better visualization of the results, the trends of the mass loss of the samples over time are shown in Figure 7.

### 3.4. Flexural and Compressive Strength

**Figure 8:**
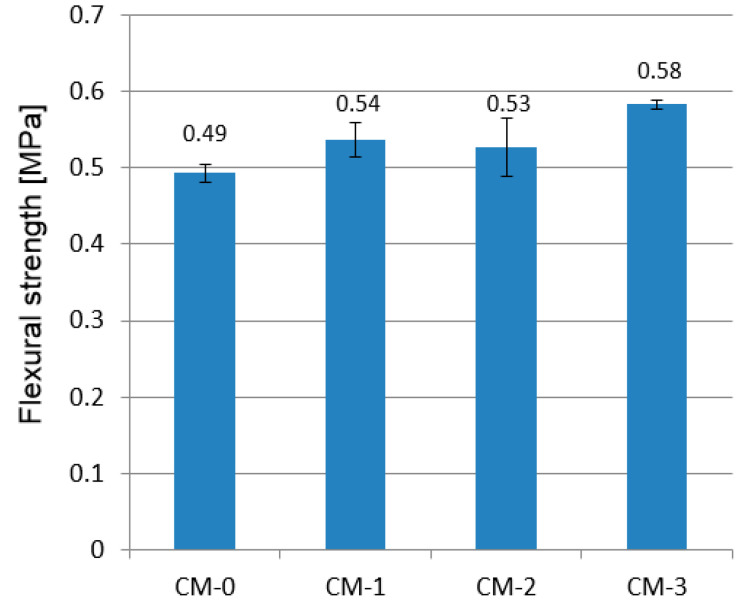
Flexural strength of tested mortars.

**Figure 9:**
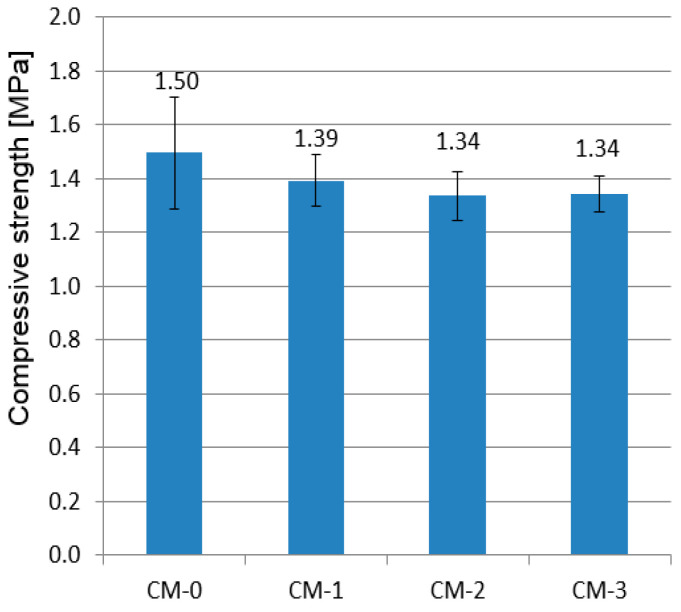
Compressive strength of tested mortars.

The results of the flexural and compressive strength tests are shown in Figure 8 and Figure 9, respectively. Error bars depict standard deviation. 

**Figure 10:**
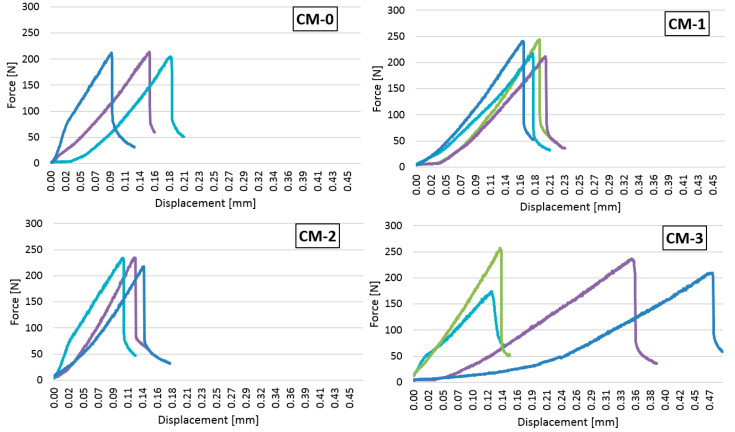
The dependence of the bending force on the displacement of the press head.

**Figure 11:**
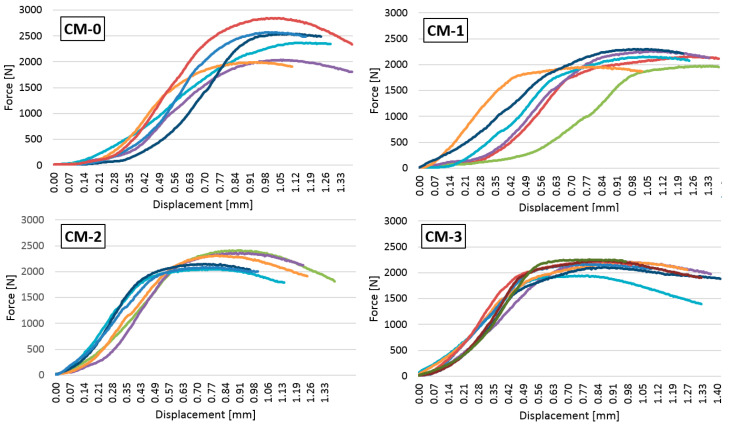
The dependence of the compressive force on the displacement of the press head.

Besides the strength of the mortars, it is also important to know how they behave under increasing loads. Figure 10 shows the dependence of the bending force on displacement of the press head, while Figure 11 shows the compressive force and displacement of the press head during the mortar strength test.

## 4. Discussion

### 4.1. Apparent Density, Specific Density, and Total Porosity

The apparent density of the tested mortars ranged between 1802 and 1853 kg/m^3^. The differences were small, and taking into account the standard deviation, it is difficult to determine the effect of the admixture of linseed oil varnish on the parameter. However, in all cases, the admixture led to an increase in bulk density. The admixture in the amount of 1% and 3% increased the mortar density by 0.2% and by 0.9%, respectively, concerning the reference sample. The admixture in the amount of 2% caused the greatest increase in bulk density (by 2.8%). The results may be influenced by the heterogeneity of sample compaction, so to draw conclusions more tests should be made. 

Total porosity ranged between 30.4 and 32.3%. The reference mortar had the highest porosity, which proves that the admixture of varnish affects the sealing of the material. The lowest porosity of the material was obtained with 2% linseed oil varnish content in the clay mass (porosity reduction by 5.9%). Additions in the amount of 1% and 3% decreased the porosity by 3.1% and 4.0%, respectively, compared to the reference sample. Other studies [23] showed that the admixture of linseed oil increased the porosity of lime mortars. Čechová, in turn, stated [25] that the admixture of linseed oil in the amount of 1% slightly reduced the porosity, while in the amount of 3% it increased the porosity of lime mortars. For a more precise assessment of the impact of porosity on the remaining properties of mortars, an analysis of the size and distribution of pores should be performed.

### 4.2. Resistance to Water

Clay is a material very susceptible to water, and as a result of its direct action, the bond strength is immediately broken. The samples without linseed varnish admixture achieved the most unfavourable result in the resistance to water test. After 1-min immersion, their weight decreased by almost half, as shown in Figure 5. For this reason, it was decided to reduce the time of their water bath to less than 1 min. As the addition of linseed varnish increases, the mortar becomes more and more resistant to water. It is evidenced by the decrease in the loss of mortar mass. As the immersion time increases, the average weight loss increases for all recipes. The best results were achieved by samples with an admixture of linseed varnish in the amount of 3% of the clay weight. After a 7-min bath, their weight decreased only by 0.8%. The samples containing 2% of the admixture were ranked second with a good result. Their mass after a 7-min immersion decreased by 3.6%. The increase in water resistance is related to the fact that a substance with hydrophobic properties was introduced into the structure of the mortar, and during mixing, it created a water barrier on the surface of the clay and sand particles. Hydrophobization with linseed varnish led to a slight reduction in total porosity, which could also reduce the penetration of water into the mortar structure. The results prove that the structural hydrophobization of clay mortars with linseed varnish brought positive effects. However, increasing the amount of dopant should be considered. Minke and Krick in their work [53] report that the addition of linseed varnish in the amount of 4 and 6% of the clay plaster volume protects the plaster surface against rain erosion for more than 6 days, while clay plaster without additives erodes after 3 s of rain activity. The mechanisms and chemical reactions that occur when the lime binder is combined with linseed oil are described in Ref. [54]. Analogous analyses of the chemical reactions occurring in the combination of linseed oil with clay are needed. In other studies [22], after using 1.5% casein and 4.5% hydrated lime to clay weight, it was possible to reduce the weight loss of clay mortar after 30 min of soaking in water to 1.9% compared to the non-soaked sample. On the other hand, the resistance of the material to the effect of water is often associated with a reduction in the vapour permeability [26,53,55], which is why further research should relate to this issue.

### 4.3. Shrinkage and Weight Loss on Drying

Considering the samples ordered according to the growing amount of linseed oil admixture (from CM-0 to CM-3), the decrease in length after 19 days of measurements amounted to 2.2%, 1.5%, 0.9%, and 0.6%, respectively, compared to the original size. As the addition of linseed varnish increased, the shrinkage decreased. Its value in the case of CM-3 mortar was reduced by 74.4% compared with the mortar without admixture. The influence of the higher admixture content on the parameter would also be worth investigating, as it is difficult to conclude on the effect of the insertion exceeding the 3% value. 

All mortars showed the highest weight loss during the first 3 days after demoulding. Later, the results remained at the same level and slight declines were recorded. As the amount of linseed varnish increased, the percentage of weight loss during drying decreased. The varnish sealed the mortar, preventing the evaporation of free water. Samples without linseed varnish admixture showed 62.5% greater weight loss compared to CM-3. The weight loss values for each recipe were similar and had a small standard deviation. After 19 days, the mass of the samples was stabilized, and no weight loss was recorded in the following days. In another study [20], the drying capacity of lime mortars with an admixture of, among others, olive oil was examined. Weight loss progressed rapidly over the first 12 days, and the weight of the samples stabilized after 15 days.

### 4.4. Flexural and Compressive Strength

The flexural strength of the tested mortars ranged between 0.49 and 0.58 MPa and the examined admixture improved the flexural strength. The additive of 1% and 2% increased the average strength of the mortars by 10.2% and 8.2%, respectively, compared with the CM-0 formula. Samples with 3% varnish content achieved the highest strength (0.58 MPa—18.4% increase) and the smallest dispersion of results. The flexural strength of CM-2 samples was lower than CM-1, but the results were characterized by the highest standard deviation, so the test should be performed on a larger number of samples for comparison.

The compressive strength of the tested mortars ranged between 1.34 and 1.50 MPa. Each of the analysed contents of the admixture deteriorated the compressive strength of the mortar, the effect being the strongest for the highest amount of the varnish. However, it is worth noting that results obtained from the reference mortar tests showed the greatest discrepancy. After using the admixture, the results within each recipe were more uniform, so varnish improved the homogeneity of the material. The reason for the decrease in strength may be the fact that the aggregate is surrounded with linseed oil varnish, and thus the bond between the clay and sand is weakened. The second reason may be the fact that the samples were not dried at high temperature before testing but under natural, air-dry conditions. As a result, looking at Figure 7, the samples containing varnish admixture maintained a higher level of moisture which could also weaken the clay-sand bond. However, these phenomena should also cause a decrease in bending strength, but this increases with the amount of admixture. The admixture of varnish slightly decreased the total porosity, but it did not improve the strength parameters. A clear relationship between the total porosity and strength of other mortars modified by the linseed oil was not noticed in other studies as well [23,24,56]. Another study [57] investigated the effect of a 2% linseed oil admixture on the compressive strength of lime mortar. The admixture of oil caused a decrease in strength by approximately 32% compared to the reference mortar. The authors indicated that it may be caused by reduced carbonation, increased porosity, and weakening of the binder–aggregate contact zone due to the presence of oil. Čechová et al. [58], on the other hand, studied the effect of linseed oil admixture on the strength of lime mortars modified with pozzolana and cement. The oil admixture in the amount of 1% led to an increase in strength, while in the amount of 3% caused a drastic decrease in mortar strength. In turn, in the case of cement mortars, Justnes et al. [56] showed that the admixture of linseed oil in the amount of 0.5–1.5 wt % caused a significant decrease in mechanical strength.

The bending force–displacement plots (Figure 10) of the samples CM-1 and CM-2 are similar and do not show large discrepancies between the samples prepared according to each recipe. A larger divergence appeared for the CM-0 mortar—the destructive force caused a displacement of about 0.08 mm to 0.17 mm. The greatest differences in the behaviour of the material under load were shown by samples based on the CM-3 formula. Although the values of the destructive force were similar, they produced a displacement ranging from 0.13 mm to 0.46 mm. The CM-2 samples showed the greatest stiffness because the destruction occurred with the smallest deformation (axial displacement in the range of 0.09–0.14 mm).

The dependence of the compressive force on the axial displacement of the compression head is shown in Figure 11. The smallest differences between the behaviour of individual samples within one recipe under increasing load were observed for the CM-2 and CM-3 recipes, where, as previously mentioned, the admixture of varnish improved the homogeneity of the mortars. The admixture of varnish in the amount of 2% and 3% improved the stiffness of the material, as the maximum compressive force (similar to the CM-1 samples) occurred with smaller deformation, in the range between 0.60 and 0.90 mm.

## 5. Conclusions

This article presents research on the properties of glauconite clay mortars differing in the amount of linseed varnish admixture. A thorough analysis of the results made is possible to formulate the following conclusions:

1. The admixture of linseed oil varnish in the amounts used in the investigation had a positive effect on some of the tested properties; regardless of the quantity of the admixture, the modified mortars had better parameters concerning flexural strength, shrinkage reduction, and water resistance than the reference mortar, without admixture;
2. The admixture of varnish increased the flexural strength of the mortars, deteriorating their compressive strength; the improvement amounted to 18.4% in the case of the samples prepared according to the CM-3 recipe;
3. As the addition of linseed varnish increased, the mortar became more and more resistant to water; after a 7-min bath, the weight of samples with an admixture of linseed varnish in the amount of 3% of the clay weight (CM-3) decreased by only 0.8%; the effect can be useful in developing recipes for external plasters;
4. The admixture of linseed varnish significantly reduced shrinkage; the best results were obtained by using the highest amount of admixture, and the shrinkage value of the CM-3 mortar was reduced by 74.4% compared with the shrinkage of the mortar without admixture;
5. The reference mortar without admixture was characterized by the highest total porosity, which proved that the addition of varnish sealed the mortar.

Currently, the authors are examining the influence of various admixtures, including linseed varnish, on the vapour permeability of clay mortars. It is an important property when the mortar is used as a plaster on walls made of organic materials, such as straw or hemp–lime composites.

## Figures and Tables

**Table 1:** Recipes of mortars (weight ratios in relation to clay)**.**

Symbol	Clay	Sand	Linseed Oil Varnish	Water
CM-0	1	4	0	0.8
CM-1	1	4	0.01	0.8
CM-2	1	4	0.02	0.8
CM-3	1	4	0.03	0.8

**Table 2:** Chemical composition of glauconite clay used in the investigation [49].

Component	(%)
SiO_2_	51.12
Fe_2_O_3_	19.00
MgO	4.74
Al_2_O_3_	6.09
CaO	2.16
Na_2_O	0.05
K_2_O	8.59
P_2_O_5_	1.27
TiO_2_	0.06

**Table 3:** Apparent density, specific density, and total porosity of the mortar (standard deviation in brackets)**.**

Mortar	Apparent Density (kg/m^3^)	Specific Density (kg/m^3^)	Total Porosity (%)
CM-0	1802.0 (±19.3)	2655.0 (±28.2)	32.3 (±0.75)
CM-1	1817.0 (±20.8)	2644.0 (±9.2)	31.3 (±0.76)
CM-2	1853.0 (±19.0)	2662.0 (±11.8)	30.4 (±0.53)
CM-3	1832.0 (±16.3)	2656.0 (±15.2)	31.0 (±0.44)

**Table 4:** The average values of the length loss of the samples as a result of shrinkage and the standard deviation.

Mortar	Days of Measurement							
	0th	3rd	6th	8th	10th	12th	15th	19th
	Length Loss/±SD (%)							
CM-0	100.00	99.69	98.32	98.32	98.32	98.30	98.01	97.78
	±0.00	±0.31	±0.36	±0.36	±0.36	±0.33	±0.18	±0.38
CM-1	100.00	99.79	99.45	98.98	98.90	98.81	98.73	98.51
	±0.00	±0.18	±0.48	±0.50	±0.43	±0.35	±0.31	±0.18
CM-2	100.00	99.79	99.49	99.40	99.46	99.23	99.10	99.10
	±0.00	±0.10	±0.13	±0.08	±0.10	±0.24	±0.36	±0.34
CM-3	100.00	99.77	99.64	99.60	99.54	99.47	99.30	99.43
	±0.00	±0.18	±0.18	±0.26	±0.20	±0.20	±0.44	±0.17

**Table 5:** The average values of the mass loss of the samples over time and the standard deviation.

Mortar	Days of Measurement							
	0th	3rd	6th	8th	10th	12th	15th	19th
	Mass Loss/±SD (%)							
CM-0	100.00	92.50	90.89	90.77	90.77	90.80	90.72	90.71
	±0.00	±0.34	±0.52	±0.51	±0.51	±0.57	±0.52	±0.52
CM-1	100.00	92.94	91.42	91.13	90.46	91.35	91.21	91.22
	±0.00	±1.01	±0.94	±1.08	±0.98	±0.97	±1.00	±0.96
CM-2	100.00	94.11	93.58	93.49	93.51	93.51	93.40	93.40
	±0.00	±0.35	±0.39	±0.46	±0.40	±0.39	±0.39	±0.40
CM-3	100.00	95.38	94.43	94.31	94.34	94.28	94.27	94.20
	±0.00	±0.51	±0.56	±0.57	±0.44	±0.55	±0.64	±0.54
